# Impact of the 23-valent pneumococcal polysaccharide vaccination in pregnancy against infant acute lower respiratory infections in the Northern Territory of Australia

**DOI:** 10.1186/s41479-018-0057-2

**Published:** 2018-12-25

**Authors:** Michael J. Binks, Sarah A. Moberley, Anne Balloch, Amanda J. Leach, Sandra Nelson, Kim M. Hare, Cate Wilson, Jane Nelson, Peter S. Morris, Robert S. Ware, Mimi L. K. Tang, Paul J. Torzillo, Jonathan R. Carapetis, Kim Mulholland, Ross M. Andrews

**Affiliations:** 1 Menzies School of Health Research, Charles Darwin University, Darwin, Northern Territory 0810 Australia; 2 Murdoch Children’s Research Institute, University of Melbourne, Royal Children’s Hospital, Melbourne, Victoria Australia; 3 Department of Health and Families, Darwin, Northern Territory Australia; 4 Menzies Health Institute Queensland, Griffith University, Gold Coast; Child Health Research Centre, School of Medicine, The University of Queensland, Queensland, Brisbane, Australia; 5 Royal Prince Alfred Hospital, Sydney, New South Wales Australia; 6 Telethon Kids Institute, University of Western Australia, Perth Children’s Hospital, Perth, Western Australia Australia; 7 National Centre for Epidemiology and Population Health, Australian National University, Canberra, Australian Capital Territory Australia

**Keywords:** 23-valent pneumococcal polysaccharide vaccine, Pregnancy, Acute lower respiratory infection, Australia, Indigenous

## Abstract

**Background:**

Indigenous children in Australia’s Northern Territory are densely colonised with the pneumococcus within weeks of birth antecedent to a high prevalence of acute lower respiratory infection (ALRI). We assessed the impact of the 23-valent pneumococcal polysaccharide vaccine (23vPPV) in pregnancy against infant ALRI in this setting.

**Methods:**

In an open label, allocation concealed, outcome-assessor blinded, randomised controlled trial conducted in the Northern Territory of Australia, healthy Indigenous women aged 17–39 years were randomised to receive the 23vPPV during pregnancy (*n* = 75; 30–36 weeks gestation), at birth (*n* = 75), or at 7 months post-partum (*n* = 77). Randomisation was stratified by community of residence. In a secondary analysis, we compared the incidence of ALRI hospitalisations and ALRI clinic presentations (ascertained from electronic medical records) among infants of pregnancy vaccinees versus infants of mothers not vaccinated in pregnancy (controls) in the first year of life.

**Results:**

ALRI hospitalisation incidence was 12.3 per 100 child-years among infants of pregnancy vaccinees compared with 15.8 per 100 child-years among controls (hazard ratio (HR) 0.77, 95%CI 0.29–2.03). ALRI hospitalisations were more common among remote compared to urban infants (27.7 versus 8.6 per 100 child-years). Stratification by dwelling highlighted a differential antenatal vaccine effect against ALRI hospitalisations (*urban* HR 2.45, 95%CI 0.60–9.99; *remote* HR 0.21, 95%CI 0.04–1.08). ALRI clinic presentation incidence was similar among infants of pregnancy vaccinees and controls.

**Conclusions:**

In this small study, antenatal 23vPPV vaccination was not associated with a reduced incidence of infant ALRI hospitalisations or ALRI clinic presentations during the first year of life. A potential differential effect between urban and remote settings warrants further investigation.

**Trial registration:**

PneuMum; ClinicalTrials.gov NCT00714064.

**Electronic supplementary material:**

The online version of this article (10.1186/s41479-018-0057-2) contains supplementary material, which is available to authorized users.

## Background

In the “PneuMum” randomised controlled trial [1], vaccine efficacy (VE) against all-cause ear disease among infants of Indigenous Australian women who had received the 23-valent pneumococcal polysaccharide vaccine (23vPPV) in pregnancy was non-significant when compared with unvaccinated controls (VE 12, 95% confidence interval (CI) -12 to 31%). A post-hoc analysis suggested the benefit of 23vPPV in pregnancy was limited to protection against infant ear disease concurrent with carriage of 23vPPV serotypes (VE 51, 95%CI -2-76%). This suggested benefit was consistent with the observed elevation in vaccine-serotype specific antibodies in maternal blood, cord blood and breast milk among pregnancy vaccines compared to controls [1].

Acute lower respiratory infections (ALRIs) are prominent among Indigenous infants in the Northern Territory [2]. Over 20% of Indigenous children are admitted to hospital [3] and 75% present to primary health care [4] with an ALRI in their first year of life. Though health care is freely available, rates of radiologically confirmed pneumonia are among the highest reported in the world [5]. Indigenous infants are densely colonised with the pneumococcus within 6 weeks of birth, prior to protection by infant vaccines, and carriage prevalence reaches 80% by 6 months of age [6]. While the aetiology of ALRI is difficult to characterise, the pneumococcus is commonly isolated from the middle ear discharge of Indigenous children with otitis media (23%) [7] and from bronchoalveolar lavages collected from Indigenous children with chronic suppurative lung disease (33%) [8]. Pragmatic strategies such as maternal vaccination are essential to reduce the burden of early-onset pneumococcal infection in this region.

In other settings (Nepal, Mali and South Africa) the inactivated influenza vaccination in pregnancy reduced the incidence of severe infant pneumonia by 20% (Incidence rate ratio 0.80, 95%CI 0.66–0.99) [9]. Three trials have previously assessed infant ALRI following maternal 23vPPV in pregnancy. In Papua New Guinea in 1973, 187 pregnant mothers received a 14-valent PPV and 167 received a saline placebo [10]; morbidity surveillance showed that antenatal vaccination had a VE of 14% (*p* = 0.100) and postnatal vaccination (when children were aged 1–17 months) had a VE of 17% (*p* = 0.020), against episodes of ALRI until 3 years of age. This was the first suggestion that maternal pneumococcal vaccination may protect infants against pneumococcal disease. In a trial of 23vPPV (*n* = 75) versus meningococcal polysaccharide vaccine (groups A + C; *n* = 75) in pregnancy conducted in The Gambia in the early 1990s [11], infants underwent one year of post-vaccination clinic surveillance. Whilst the frequency was low, there were half as many pneumonia episodes among infants of 23vPPV vaccinees (*n* = 4) compared to control infants (*n* = 8; meningococcal vaccinees). More recently in 2005 and 2006, a Brazilian trial of 23vPPV in pregnancy [12] examined infants and questioned mothers monthly to identify the occurrence of acute respiratory infections during the first 6 months of life. There was no difference in the prevalence of acute respiratory infections between infants of vaccinated and unvaccinated mothers at 3 (8.5% versus 8.5%) or 6 (25.5% versus 29.8%) months of age. Ultimately, ALRI outcome data are sparse in maternal pneumococcal vaccination studies globally. A Cochrane review (first published in 2006 and last updated in 2015) included clinical outcomes from only the two latter studies above (*n* = 241) and concluded that more evidence is required to support the use of 23vPPV vaccination in pregnancy for reducing infant infections [13].

Given the endemicity of pneumococcal carriage and ALRI in the Northern Territory, we hypothesised that maternal 23vPPV vaccination would reduce the rate of infant ALRI episodes in the first year of life. In this secondary analysis of the PneuMum randomised controlled trial, we assessed maternal 23vPPV vaccine efficacy against ALRI episodes among high-risk Australian Indigenous infants.

## Methods

### Study design

This was a secondary analysis of an open label, allocation concealed, outcome-assessor blinded, randomised controlled trial of maternal 23vPPV (PNEUMOVAX® 23, Merck, USA) in pregnancy. The trial was conducted between August 2006 and January 2011. The detailed trial design, eligibility, randomisation procedures (stratified by community of residence) and co-primary outcomes (middle ear disease and infant pneumococcal carriage) are described elsewhere [1]. The original study protocol is available online [14].

### Participants

Healthy pregnant Indigenous women (aged 17–39 years) and their infants living in Darwin or remote Northern Territory communities of Australia who intended to remain in the study catchment area for the duration of the study.

### Intervention

Mothers were randomised (1:1:1) to receive the 23vPPV during the third trimester of pregnancy (group 1 = 75; 30–36 weeks gestation), at birth (group 2 = 75), or at the final study visit 7 months post-partum (group 3 = 77; offered at study exit).

**Fig. 1:**
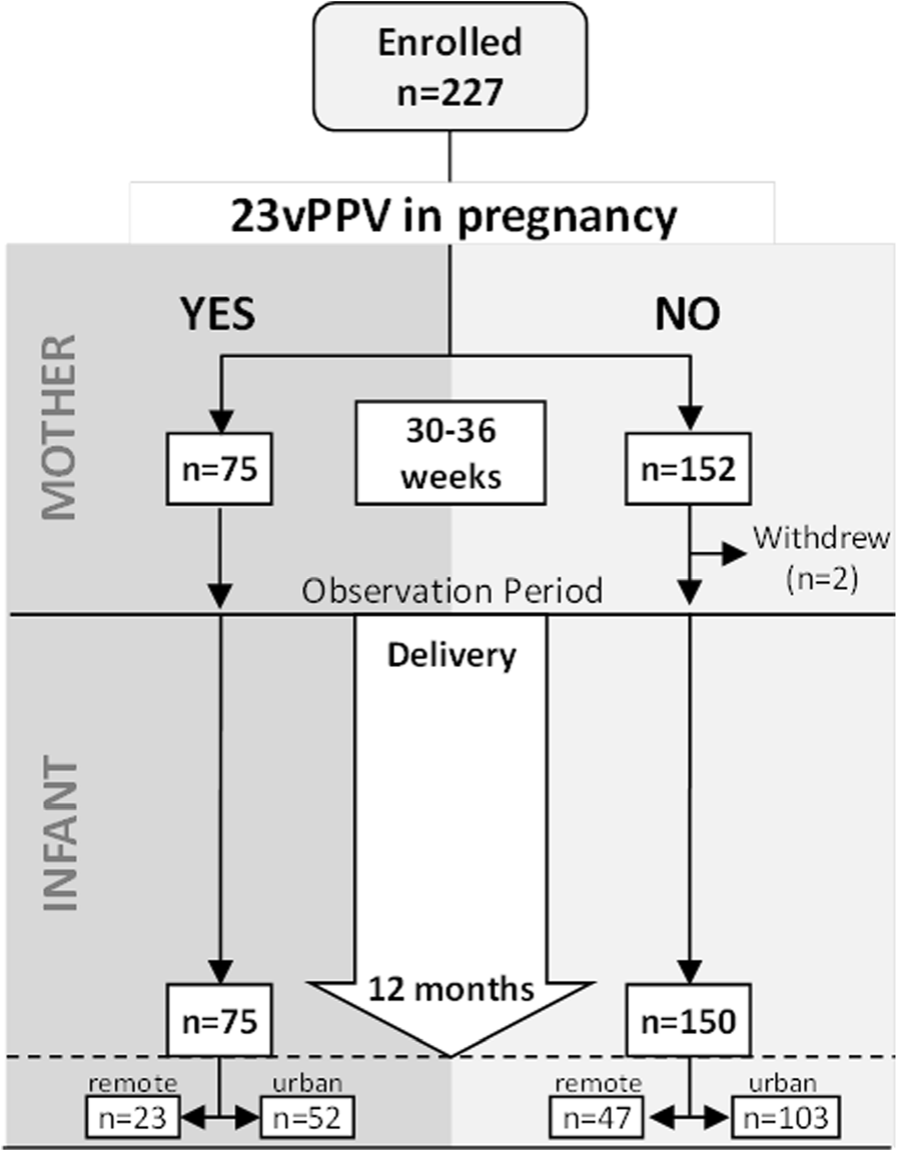
Study participant flow diagram. Hospital records were investigated for all 225 infants born during the study. Clinic presentations were investigated among the 70 infants from remote communities for whom electronic medical records were available

As there was no significant difference in the primary trial outcomes (infant pneumococcal carriage and/or ear disease) between groups 2 and 3 [1], we elected prior to the collection of ALRI data to assess participants in two groups (Fig. 1):

Pregnancy vaccinees-23vPPV during pregnancy (group 1)

Controls - no 23vPPV during pregnancy (groups 2 and 3 combined)

### Outcomes

The outcomes of interest for this analysis were ALRI hospitalisations and ALRI clinic presentations. Blinded assessors reviewed infant hospital and primary-care clinic medical records between birth and age 12 months. Follow-up was extended to infant age 12 months to capture maternal vaccine effects both before and after the routine infant pneumococcal conjugate vaccine (PCV) schedule recommended at 2, 4 and 6 months of age.

ALRI hospitalisation episodes were identified by International Classification of Diseases (10th Edition, Australian modification; ICD-10-AM) coding J09-J22 and A37-A37.9 [15] recorded electronically in the Northern Territory-wide government hospital discharge dataset. Data were extracted by the Acute Care Information Unit at Royal Darwin Hospital. ALRI clinic presentations (remote participants only) were identified by applying a standardised diagnostic algorithm to medical records accessed via the electronic Primary Care Information System (PCIS). Guided by previous work [16], ALRI clinic presentations were defined by at least two primary indicators: ALRI diagnosis in clinic notes, chest recession, tachypnoea (<2mo: ≥60 breaths per minute; 2-12mo: ≥50 breaths per minute) or cough (any); or one of these primary indicators and at least two secondary indicators including: wheeze, crackles, fever (≥38 °C) or specific treatment with at least 2 days of enteral antibiotics. Any hospitalisations (identified by any ICD-10-AM hospital coding) and any clinic presentations (identified by any non-scheduled presentation recorded in PCIS) were also documented for comparison. An episode was defined as unique with at least 14 days of separation following hospital discharge or leaving the clinic. Hospitalisations and clinic presentations within 7 days of birth were excluded from analysis to avoid episodes associated with birth.

### Study power

We expected 22% of Indigenous children to be hospitalised with an ALRI before age 12 months [3]. Given the sample size was known (*n* = 75 in the intervention group and *n* = 150 in the control group), we were powered (80%; α = 0.05) to detect a 2.5-fold or greater reduction in infant ALRI hospitalisations between groups. We expected 75% of Indigenous children to present to their primary health care clinic before age 12 months [4]. As PCIS electronic clinic data were only available for the 70 remote infants (*n* = 23 in the intervention group and *n* = 47 in the control group), we were powered (80%; α = 0.05) to detect a 2-fold or greater reduction in infant ALRI clinic presentations between groups.

### Analysis

Analysis was performed on an intention to treat basis. Incidence rates for ALRI hospitalisation (all infants) and ALRI clinic presentation (remote infants only) were calculated by dividing the number of ALRI events by the time-at-risk and under observation for each infant during the period from birth to 12 months of age. Time-at-risk excluded birth (7 days) and ALRI episode (14 days) washout periods. For hospitalisation episodes the length of hospital stay was also excluded.

For the main analysis we compared the hazard rates of ALRI episodes between pregnancy vaccinees and controls using univariate and multivariate Cox proportional hazard models with shared frailty, presenting effect estimates as a hazard ratio (HR) and 95%CI. HR < 1 indicated a lower event risk among 23vPPV vaccinees and confidence intervals excluding 1 were considered statistically significant. Covariates were selected based on their likely prognostic influence on the ALRI outcomes: remote/urban dwelling (0/1), influenza vaccination in pregnancy (0/1), duration of any breast feeding (time dependent, 1, 2 or 7 months), antenatal/postnatal maternal smoking (0/1; antenatal – reported during pregnancy; postnatal - reported at ≥2 of the 3 study visits) and number of PCV doses (time dependent; 14 days was added to each PCV dose date to allow for the immune response). Infants were scheduled to receive a 3-dose primary PCV series at 2, 4 and 6 months of age. Univariate analyses and forward and backward stepwise estimation of covariates (exclusion set at a *p*-value ≥0.25) informed the final adjusted model. Proportional-hazards assumptions were tested using log-log plots and post-model Schoenfeld residuals. Missing data were imputed for modelling; using linear interpolation for dates and logistic regression for binary outcomes. Failure curves were constructed for first ALRI episodes and the equality of survivor functions was assessed using the log-rank test. Any cause hospitalisations and clinic presentations were presented for comparison. Data analysis was performed using STATA statistical software, version 15 (StataCorp, USA).

## Results

### Participant characteristics

From 227 randomised mothers, 225 infants (two pre-birth withdrawals) were eligible for follow-up of ALRI outcomes (Fig. 1): 75 infants in the antenatal 23vPPV group and 150 in the control group. All 225 births were identified in the hospital records; 217 at Royal Darwin Hospital, six at Alice Springs Hospital and two at Darwin Private Hospital. Participant characteristics were similar between groups (Table 1). In both the antenatal 23vPPV group and controls, uptake of the seasonal influenza vaccine during pregnancy was low (12 and 21% respectively). Most infants (85%) completed their 3-dose primary PCV schedule (2, 4 and 6 months) before 12 months of age (median age 197 days). The 23vPPV was received by all 75 mothers during pregnancy as randomised. For the mothers not randomised to 23vPPV in pregnancy, 74 received 23vPPV at birth (as randomised) and 57/76 received the 23vPPV when offered at the 7 month visit (the original study exit; as randomised). Within the antenatal 23vPPV group the median time between receipt of the 23vPPV in pregnancy and birth was 6 weeks (range 1–10 weeks); four antenatal vaccinees received their dose within two weeks of birth.

**Table 1:** Participant characteristics

		23vPPV in Pregnancy	
Maternal characteristics at randomisation		Yes (*n* = 75)	No (*n* = 150)
		*median(range)*	
Maternal age, years		23 (17–39)	24 (17–38)
Household occupancy, people		4 (1–12)	4 (2–15)
Household occupancy (< 5 years)^a^, people		1 (0–4)	1 (0–8)
		*n(%)*	
Primigravida		29 (39)	50 (33)
Completed year 10^b^		61 (82)	125 (84)
Remote community residence		23 (31)	47 (31)
Smoker		36 (48)	68 (45)
Influenza vaccine in pregnancy		9 (12)	30 (20)
*Infant characteristics*			
		*median(range)*	
Gestational age at birth, weeks		39 (35–41)	39 (34–42)
Birthweight, grams		3293 (2060–4425)	3338 (2080–4620)
		*n(%)*	
Male		36 (48)	84 (56)
Low birth weight (< 2500 g)		7 (9)	8 (5)
Premature birth (< 37 weeks)		7 (9)	6 (4)
Special or intensive care admission		14 (19)	31 (21)
Breast fed^c^	*1 month study visit*	61 (92)	106 (82)
	*2 month study visit*	55 (90)	97 (77)
	*7 month study visit*	43 (64)	85 (63)
Mother smoking^d^	*1 month study visit*	35 (53)	63 (49)
	*2 month study visit*	34 (57)	68 (54)
	*7 month study visit*	46 (69)	76 (56)
3 doses of PCV < 12 months		64 (85)	127 (85)
*7vPCV*		38 (51)	78 (52)
*10vPHiD-CV*		15 (20)	34 (23)
*Mixed schedule (7v/10v)*		11 (15)	15 (10)
		*median(range)*	
Age in days at PCV1		64 (41–240)	63 (51–128)
Age in days at PCV2		130 (103–311)	132 (107–253)
Age in days at PCV3		197 (150–349)	197 (168–333)

PneuMum study vaccinations, sample collection and clinical examinations occurred over 5 visits: pregnancy (30–36 weeks), birth (< 72 h) and infant age 1, 2 and 7 months. Complete data were not available for some characteristics. For pregnancy vaccinated, and non-pregnancy vaccinated groups respectively the denominators for ^a^household occupancy (< 5 years) were: 75 and 149; for ^b^completed year 10 were: 74 and 149; for ^c^breast feeding (non-exclusive) were: 66 and 129 at 1 month; 61 and 126 at 2 months; and 67 and 134 at 7 months and for ^d^maternal smoking were: 66 and 129 at 1 month; 60 and 127 at 2 months; and 67 and 135 at 7 months. PCV: pneumococcal conjugate vaccine; includes both the 7vPCV and 10vPHiD-CV. Where PCV dose dates were missing or outside the 12 month (−14 days) window the dose was coded as not received

### Episodes of care

By 12 months of age, 27% (60/225) of infants were admitted to hospital for 84 episodes of care and 11% (25/225) with an ALRI for 32 episodes of care. The respective median ages of first hospitalisation (any cause) and first ALRI hospitalisation, were 98 days (range 9 to 347) and 155 days (range 13 to 346). Over the 12 month observation period, hospitalisations were more frequent among remote compared to urban dwelling infants: any cause (40%; 28/70 versus 21%; 32/155) and ALRI (21%; 15/70 versus 6%; 10/155). By 12 months of age, all 70 remote dwelling infants had presented to their medical clinic for 459 episodes of care and 74% (52/70) with an ALRI for 126 episodes of care. For the remote dwelling infant group, the respective median ages of first clinic presentation (any cause) and ALRI clinic presentation, were 48 days (range 10 to 317) and 148 days (range 36 to 364).

### ALRI hospitalisations in the antenatal 23vPPV group and controls

The proportion of children presenting to hospital with an ALRI before 12 months of age was 8% (6/75) in the antenatal 23vPPV group and 13% (19/150) in the control group (Table 2). Among the antenatal vaccinees, 5 of the 6 ALRI hospitalised children had mothers who were vaccinated within 6 weeks (the median) of birth; only one of these was born premature (< 37 weeks gestation) and small numbers limited further interpretation. One infant in the antenatal 23vPPV group had four ALRI hospitalisation episodes and one in the control group had five episodes. No other infants had multiple ALRI hospitalisation episodes. There were no significant differences in the length of hospital stay, or time between ALRI hospitalisation episodes, among infants in the antenatal 23vPPV group compared to the control group.

**Table 2:** ALRI episodes among infants (age < 12 months) of pregnancy vaccinees and controls

		ALRI hospitalisations		ALRI clinic presentations	
		23vPPV in Pregnancy		23vPPV in Pregnancy	
		Yes (*n* = 75)	No (*n* = 150)	Yes (*n* = 23)	No (*n* = 47)
Children	n (%)	6 (8)	19 (13)	18 (78)	34 (72)
Episodes	n	9	23	45	81
Episodes per child*	median (range)	1 (1–4)	1 (1–5)	2.5 (1–6)	2 (1–7)
Multiple episodes*	n (%)	1 (17)	1 (5)	13 (72)	18 (53)
Length of stay in days	median (range)	3 (1–7)	3 (1–39)	na	na
Days between episodes^#^	median (range)	28 (23–211)	66 (35–105)	64 (18–182)	46 (16–220)

*per presenting child. ^#^Among children with multiple episodes. na: not applicable

Overall, the 32 ALRI hospitalisation episodes occurred during 219.0 years of at risk observation. The incidence of infant ALRI hospitalisation events (Table 3) was 12.3 per 100 child-years in the antenatal 23vPPV group compared to 15.8 per 100 child-years in the control group (hazard ratio (HR) 0.77, 95%CI 0.29–2.03). In a multivariate model adjusting for remote dwelling, breast feeding duration and maternal smoking in pregnancy the adjusted hazard rato (aHR) for infant ALRI hospitalisation events in the antenatal 23vPPV group versus controls was 0.92, 95%CI 0.34–2.47. In this model remote dwelling was significantly associated with ALRI hospitalisation (aHR 3.16, 95%CI 1.26–7.93) while breast feeding duration and maternal smoking in pregnancy had no significant influence. Eleven missing breast feeding stop dates were interpolated. There was little evidence of a hazard bias due to PCV dosing or timeliness with respect to ALRI hospitalisation outcomes according to 23vPPV receipt (Additional file 1: Table S1) and this variable was omitted from the final model during stepwise covariate selection. Incidence of any cause hospitalisation (36 versus 40 episodes per 100 child-years) was consistent among infants in the antenatal 23vPPV and control groups respectively. All modelled data fulfilled the criteria for Cox proportional hazards analysis.

**Table 3:** Incidence of ALRI hospitalisation and clinic presentation episodes among infants (age < 12 months) of pregnancy vaccinees versus controls

	23vPPV in Pregnancy									
	Yes (*n* = 75)				No (*n* = 150)					
	Children, n (%)	Episodes, n	Child-years	IR	Children, n (%)	Episodes, n	Child-years	IR	HR (95%CI)	aHR (95%CI)
ALRI hospital	6 (8)	9	73.1	12.3	19 (13)	23	145.9	15.8	0.77 (0.29,2.03)	0.92 (0.34,2.47)
ALRI clinic^#^	18 (78)	45	20.9	215	34 (72)	81	43.0	188	1.13 (0.67,1.93)	1.24 (0.76,2.03)

*ALRI* acute lower respiratory infection, *IR* incidence rate per 100 child-years. *HR* hazard ratio, *aHR* adjusted hazard ratio, *95%CI* 95% confidence interval. After stepwise elimination the covariates: remote dwelling, maternal smoking in pregnancy and breast feeding duration (time dependent) were included in the final ALRI hospital model; missing breast feeding duration data (*n* = 11) were imputed. After stepwise elimination the covariates: PCV doses (time dependent), breast feeding duration (time dependent) and maternal smoking during infancy were included in the final ALRI clinic presentation model. ^#^ALRI clinic presentation data were only available for the 70 infants from remote communities (23vPPV *n* = 23; no 23vPPV *n* = 47); missing maternal smoking during infancy data (*n* = 7) were imputed

### ALRI hospitalisations in the antenatal 23vPPV group and controls stratified by dwelling

For urban dwelling infants, the incidence of ALRI hospitalisation was higher in the antenatal 23vPPV group compared to the control group (HR 2.45, 95%CI 0.60 to 9.99) while for remote infants the incidence of ALRI hospitalisation was lower in the antenatal vaccinees compared to controls (HR 0.21, 95%CI 0.04 to 1.08) and approaching statistical significance (Table 4). This phenomenon was also evident for all-cause hospitalisations but to a lesser extent.

**Table 4:** Incidence of hospitalisation episodes among infants (age < 12 months) of pregnancy vaccinees versus controls, stratified by dwelling

		23vPPV in Pregnancy								
		Yes (*n* = 75)				No (*n* = 150)				
		*urban n = 52, remote n = 23*				*urban n = 103, remote n = 47*				
Hospital episodes		Children n (%)	Episodes n	Child-years	IR	Children n (%)	Episodes n	child-years	IR	HR (95%CI)
Any cause	*Urban*	12 (23)	18	50.2	36	20 (19)	28	99.7	28	1.27 (0.57,2.86)
	*Remote*	7 (30)	8	22.2	36	21 (45)	30	44.5	67	0.52 (0.22,1.24)
ALRI	*Urban*	4 (8)	7	50.7	14	6 (6)	6	100.8	6	2.45 (0.60,9.99)
	*Remote*	2 (9)	2	22.4	9	13 (28)	17	45.1	38	0.21 (0.04,1.08)

*IR* incidence rate per 100 child-years, *HR* hazard ratio

### Time to first ALRI

**Fig. 2:**
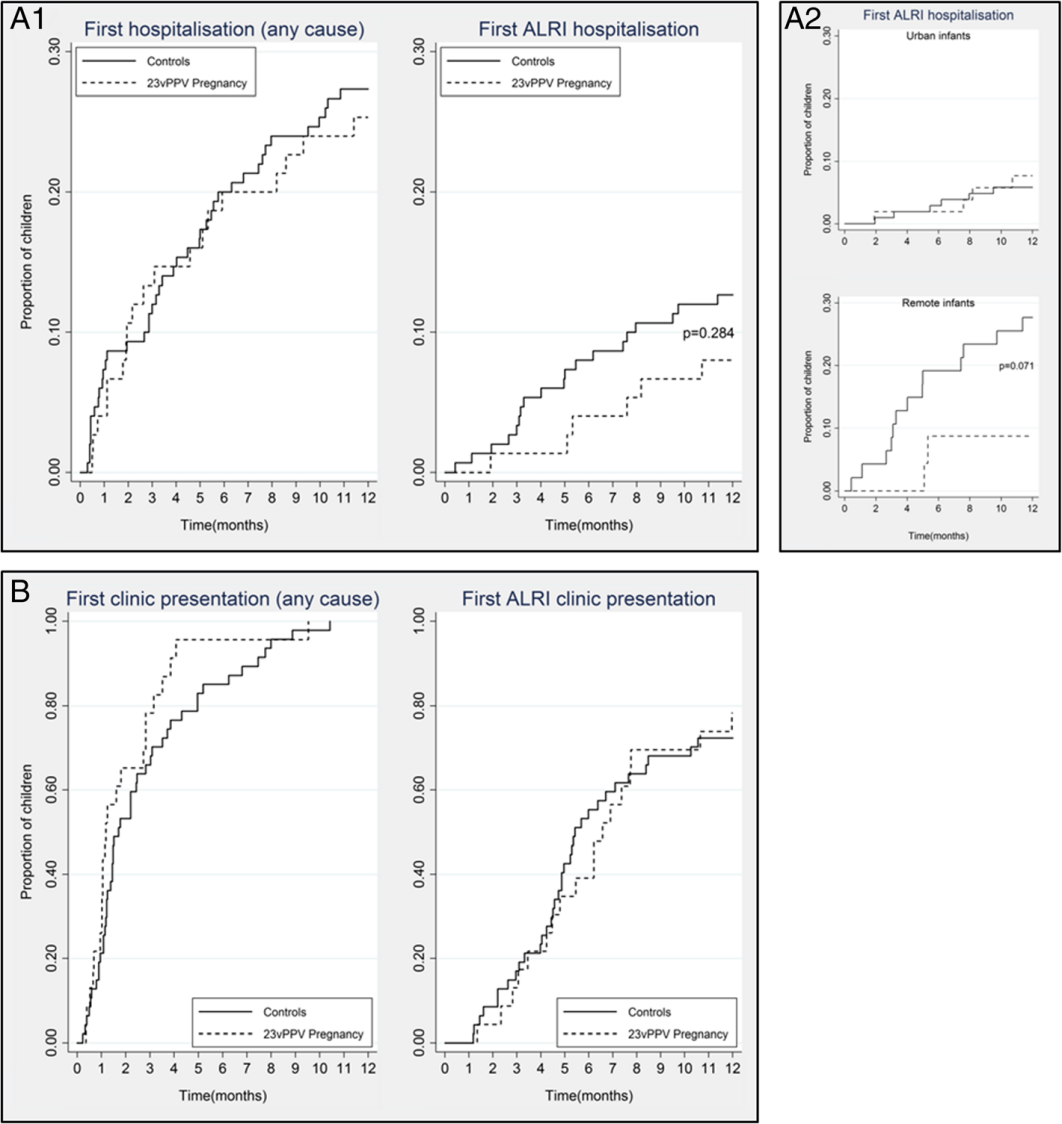
Time to first ALRI episodes. Cumulative proportion of first hospitalisations between birth and 12 months of age for infants of pregnancy vaccinees (dashes) compared to controls (solid lines). Any and ALRI hospitalisation (A1; overall) (A2; by dwelling). Any and ALRI clinic presentations (B; data available for the 70 remote participants only). The logrank test was used to indicate the equality of the Kaplan-Meier failure functions

There was a delay in time to first ALRI hospitalisation among infants of antenatal vaccinees compared to the controls (Fig. 2a1) but the inequality of the Kaplan-Meier failure functions was not statistically significant (*p* = 0.284). Stratification by dwelling (Fig. 2a2) showed that any potential vaccine effect occurred predominantly among remote participants where the difference in failure functions approached statistical significance (*p* = 0.071). There was no statistically significant difference in the time to first hospitalisation of any cause between groups (Fig. 2a1).

### ALRI primary health care presentations in remote communities

Among the 70 remote children, the proportion presenting to primary health care with an ALRI before 12 months of age was 78% (18/23) in the antenatal 23vPPV group and 72% (34/47) in the control group. Overall, the 126 ALRI clinic presentation episodes (70 remote participants only) occurred during 63.9 years of at risk observation. The incidence of ALRI clinic presentation (Table 3) was 215 per 100 child-years among infants of antenatal vaccinees compared to 188 per 100 child-years among infants in the control group (HR 1.13, 95%CI 0.67–1.93). In a multivariate model adjusting for PCV doses, breast feeding duration and maternal smoking in infancy the aHR was 1.24, 95%CI 0.76–2.03. Seven missing data points for maternal smoking were imputed (10 imputations). There was no significant difference in the incidence of any cause clinic presentation (904 versus 889 per 100 child-years) nor in time to ALRI or any cause clinic presentation between groups (Fig. 2b).

## Discussion

We did not observe a clinically or statistically significant effect of 23vPPV in the third trimester of pregnancy against the incidence of infant ALRI hospitalisation episodes or clinic presentations during the first year of life. Fewer infants than expected were hospitalised with an ALRI before 12 months of age (expected 22%, actual 11%) which impacted the statistical power of our planned analysis.

Our study suggests that any 23vPPV effect may be limited to remote settings where the burden of pneumococcal ALRI is greatest. Remote dwelling had a positive association with ALRI hospitalisation incidence and though our multivariate Cox model did not support a vaccine impact across the whole study cohort, subgroup analyses indicated that antenatal 23vPPV vaccination resulted in a lower ALRI hospitalisation rate and a delayed time to first ALRI hospitalisation among remote but not urban dwelling infants.

Given the diversity of circulating respiratory pathogens in this region, targeted strategies such as pneumococcal vaccination may reduce pneumococcal infection yet have only a limited impact against overall mucosal respiratory disease. In our primary trial findings [1], nasopharyngeal carriage of pneumococcus was detected in 62%, nontypeable *Haemophilus influenzae* in 53% and *Moraxella catarrhalis* in 38% of infants at 7 months of age. All of these bacteria are individually capable of causing both upper and lower respiratory disease. Further, respiratory viruses are historically detected in the nasopharynx of up to 90% of Northern Territory Indigenous children from 6 months to 2 years of age [17]. To enhance the statistical power of existing maternal 23vPPV studies there is scope for pooled data meta-analysis; however, to further investigate the effect of pneumococcal vaccines against ALRI among Indigenous children, larger trials focussing on high ALRI-risk communities across northern Australia or elsewhere are required.

In small trials such as this, specific outcome measures are the key to an accurate conclusion. Unfortunately, pneumococcal aetiology was poorly captured in the medical records. Further, the microbiology component of the PneuMum trial was confined to 1, 2 and 7 month visits in conjunction with the infant ear exams (co-primary outcomes) and was not able to be meaningfully linked with the ALRI presentations or hospitalisations. For the 70 remote participants, the clinic presentation algorithm was designed to detect all potential acute infections related to the lower airways and we anticipated an improvement in power to detect a vaccine effect. The absence of an observed effect against ALRI clinic presentations among remote Indigenous infants might reflect a high proportion of non-pneumococcal ALRIs in the community, while the small suggestive effect against ALRI hospitalisations, should it be real, might be an indication that more severe ALRIs are, at least in part, of pneumococcal aetiology.

The original PneuMum trial was the first maternal vaccine trial to be conducted in Australia’s Northern Territory and demonstrated that such trials are possible with appropriate community engagement and dedicated staff, achieving 50% consent and almost 90% retention in this high-risk setting [18]. As ALRI was not the primary outcome, limitations of this analysis include the finite sample size and resulting lack of statistical power, the possibility that some participants moved away from the study catchment area and were unknowingly missed in follow-up, the non-specificity of the outcome (no confirmed aetiology) and the grouping of two of the randomisation groups (those vaccinated at birth and those offered the vaccine at 7 months post-partum). In those mothers receiving the 23vPPV during pregnancy, vaccine specific IgG was actively transported across the placenta directly into the foetal circulation whilst in utero. Previous studies have demonstrated that vaccine specific IgG can persist for up to 5 months in the infant after birth [11, 19, 20]. While mothers vaccinated in pregnancy were able to pass on vaccine antibodies directly via the placenta and indirectly via their breast milk, birth vaccinees (and to a lesser extent those vaccinated at 7 months if breast feeding thereafter) only had the opportunity to pass on their vaccine antibodies via breast milk. Interestingly, antenatal vaccinees generated higher concentrations of vaccine serotype specific breast milk IgA at 1, 2 and 7 months post-partum than mothers vaccinated at birth [1].

## Conclusions

In the Northern Territory, the strategy of giving the 23vPPV in pregnancy continues to hold some potential due to the early burden of pneumococcal disease and the high carriage of several 23vPPV non-conjugate vaccine serotypes, such as 15B, 33F and 10A which are also prominent local causes of invasive disease [21]. We also know that maternal 23vPPV vaccination generates a robust maternal antibody response and may protect against vaccine-type otitis media [1]. In this study, our data did not support the use of maternal 23vPPV vaccination to protect against ALRI among Indigenous infants in the Northern Territory but we had only sufficient power to detect large effects. A suggestive but non-statistically significant impact against ALRI hospitalisations in the remote setting warrants further investigation.

## Additional file

Additional file 1: **Table S1.** Timeliness of the routine infant PCV. (DOCX 23 kb)

## Abbreviations

10vPHiD-CV: 10-valent pneumococcal + *Haemophilus influenze* protein D – conjugate vaccine
23vPPV: 23-valent pneumococcal polysaccharide vaccine
7vPCV: 7-valent pneumococcal conjugate vaccine
aHR: adjusted hazard ratio
ALRI: acute lower respiratory infection
CI: confidence interval
HR: hazard ratio
ICD-10-AM: international classification of diseases, 10th Edition, Australian modification
IR: incidence rate
n: number
PCIS: primary care information system
PCV: pneumococcal conjugate vaccine
VE: vaccine efficacy

## References

[1] Binks MJ, Moberley SA, Balloch A, Leach AJ, Nelson S, Hare KM (2015). PneuMum: impact from a randomised controlled trial of maternal 23-valent pneumococcal polysaccharide vaccination on middle ear disease amongst indigenous infants, Northern Territory. Australia Vaccine.

[2] Stubbs E, Hare K, Wilson C, Morris P, Leach AJ (2005). Streptococcus pneumoniae and noncapsular Haemophilus influenzae nasal carriage and hand contamination in children: a comparison of two populations at risk of otitis media. Pediatr Infect Dis J.

[3] O'Grady KA, Torzillo PJ, Chang AB (2010). Hospitalisation of indigenous children in the Northern Territory for lower respiratory illness in the first year of life. Med J Aust.

[4] Kearns T, Clucas D, Connors C, Currie BJ, Carapetis JR, Andrews RM (2013). Clinic attendances during the first 12 months of life for aboriginal children in five remote communities of northern Australia. PLoS One.

[5] O'Grady KA, Taylor-Thomson DM, Chang AB, Torzillo PJ, Morris PS, Mackenzie GA (2010). Rates of radiologically confirmed pneumonia as defined by the World Health Organization in Northern Territory indigenous children. Med J Aust.

[6] Leach AJ, Boswell JB, Asche V, Nienhuys TG, Mathews JD (1994). Bacterial colonization of the nasopharynx predicts very early onset and persistence of otitis media in Australian aboriginal infants. Pediatr Infect Dis J.

[7] Leach AJ, Wigger C, Beissbarth J, Woltring D, Andrews R, Chatfield MD (2016). General health, otitis media, nasopharyngeal carriage and middle ear microbiology in Northern Territory aboriginal children vaccinated during consecutive periods of 10-valent or 13-valent pneumococcal conjugate vaccines. Int J Pediatr Otorhinolaryngol.

[8] Hare KM, Grimwood K, Leach AJ, Smith-Vaughan H, Torzillo PJ, Morris PS, et al. Respiratory Bacterial Pathogens in the Nasopharynx and Lower Airways of Australian Indigenous Children with Bronchiectasis. J Pediatr. 2010. 10.1016/j.jpeds.2010.06.002 20656297

[9] Omer SB, Clark DR, Aqil AR, Tapia MD, Nunes MC, Kozuki N (2018). Maternal influenza immunization and prevention of severe clinical pneumonia in young infants: analysis of randomized controlled trials conducted in Nepal, Mali and South Africa. Pediatr Infect Dis J.

[10] Riley ID, Tarr PI, Andrews M, Pfeiffer M, Howard R, Challands P (1977). Immunisation with a polyvalent pneumococcal vaccine. Reduction of adult respiratory mortality in a New Guinea highlands community. Lancet.

[11] O'Dempsey TJ, McArdle T, Ceesay SJ, Banya WA, Demba E, Secka O (1996). Immunization with a pneumococcal capsular polysaccharide vaccine during pregnancy. Vaccine.

[12] Lopes CR, Berezin EN, Ching TH, Canuto Jde S, Costa VO, Klering EM (2009). Ineffectiveness for infants of immunization of mothers with pneumococcal capsular polysaccharide vaccine during pregnancy. Braz J Infect Dis.

[13] Chaithongwongwatthana S, Yamasmit W, Limpongsanurak S, Lumbiganon P, DeSimone JA, Baxter JK (2012). Pneumococcal vaccination during pregnancy for preventing infant infection. Cochrane Database Syst Rev.

[14] Binks MJ, Moberley SA, Balloch A, Leach AJ, Nelson S, Hare KM, et al. The PneuMum Trial Protocol: http://www.menzies.edu.au/icms_docs/213758_PneuMum_Protocol.pdf. Menzies School of Health Research. 2010 (last update).

[15] National Centre for Classification in Health. The International Statistical Classification of Diseases and Related Health Problems, 10th Revision, Australian Modification (ICD-10-AM), 6th edition. National Centre for Classification in Health, Faculty of Health Sciences, The University of Sydney. 2009.

[16] Valery PC, Morris PS, Byrnes CA, Grimwood K, Torzillo PJ, Bauert PA (2013). Long-term azithromycin for indigenous children with non-cystic-fibrosis bronchiectasis or chronic suppurative lung disease (bronchiectasis intervention study): a multicentre, double-blind, randomised controlled trial. Lancet Respir Med.

[17] Binks MJ, Cheng AC, Smith-Vaughan H, Sloots T, Nissen M, Whiley D (2011). Viral-bacterial co-infection in Australian indigenous children with acute otitis media. BMC Infect Dis.

[18] Dunbar M, Moberley S, Nelson S, Leach AJ, Andrews R (2007). Clear not simple: an approach to community consultation for a maternal pneumococcal vaccine trial among indigenous women in the Northern Territory of Australia. Vaccine.

[19] Lehmann D, Pomat WS, Combs B, Dyke T, Alpers MP (2002). Maternal immunization with pneumococcal polysaccharide vaccine in the highlands of Papua New Guinea. Vaccine.

[20] Munoz FM, Englund JA, Cheesman CC, Maccato ML, Pinell PM, Nahm MH (2001). Maternal immunization with pneumococcal polysaccharide vaccine in the third trimester of gestation. Vaccine.

[21] Krause V, Cook H. Downs and ups of invasive pneumococcal disease (IPD) amid vaccine introductions in the Indigenous population of the Northern Territory (NT), Australia. 8th International Symposium on Pneumococci and Pneumococcal Diseases. Iguacu Falls, Brazil2012.

