# Supplementary material for: Impact of the 23-valent pneumococcal polysaccharide vaccination in pregnancy against infant acute lower respiratory infections in the Northern Territory of Australia
Source: Pneumonia (Nathan). 2018 Dec 25;10:13. doi: 10.1186/s41479-018-0057-2 (PMC6305569; doi:10.1186/s41479-018-0057-2)
Supplement: Supplementary file 1 — Table S1. Timeliness of the routine infant PCV. (DOCX 23 kb) [file 41479_2018_57_MOESM1_ESM.docx]

Table S1 Timeliness of the routine infant PCV.

|  |  | **ALRI hospitalisation** | | | **No ALRI hospitalisation** | | |
| --- | --- | --- | --- | --- | --- | --- | --- |
|  |  | **23vPPV in pregnancy** | | | **23vPPV in pregnancy** | | |
|  |  | **Yes**  (n=6) | **No**  (n=19) | ***p*** | **Yes**  (n=69) | **No**  (n=131) | ***p*** |
| PCV dose 1 | n (%) | 6 (100) | 18 (95) | 0.566 | 66 (96) | 129 (98) | 0.224 |
|  | Median days  (range) | 69  (55,192) | 64  (57,113) | 0.483 | 64  (41,240) | 64  (51,224) | 0.927 |
| PCV dose 2 | n (%) | 6 (100) | 18 (95) | 0.566 | 63 (91) | 124 (95) | 0.361 |
|  | Median days  (range) | 134  (124,234) | 136  (121,253) | 0.664 | 129  (103,312) | 131  (107,345) | 0.912 |
| PCV dose 3 | n (%) | 5 (83) | 17 (89) | 0.687 | 59 (86) | 110 (84) | 0.775 |
|  | Median days  (range) | 198  (184, 209) | 191  (178,281) | 0.875 | 201  (150,349) | 194  (168,333) | 0.279 |

Pneumococcal conjugate vaccination timeliness (2, 4 and 6 month schedule) between birth and 12 months of age for infants of pregnancy vaccinees compared to controls, by ALRI hospitalisation status. Infants with a missing vaccine dose date or a vaccine dose date >365 (-14) days were considered to have missed that dose during the time at risk. ***p***: Chi-square and Wilcoxon ranksum tests were used to compare proportions and non-parametric continuous data respectively.
